# How language modulates color perception in a brain-constrained deep neural network

**DOI:** 10.1016/j.isci.2026.114832

**Published:** 2026-01-29

**Authors:** Rosario Tomasello, Kai Shaman, Fynn R. Dobler, Friedemann Pulvermüller

**Affiliations:** 1 Brain Language Laboratory, Department of Philosophy and Humanities, WE4 Freie Universität Berlin, 14195 Berlin, Germany; 2 Cluster of Excellence' Matters of Activity. Image Space Material’, Humboldt Universität zu Berlin, 10099 Berlin, Germany; 3 Berlin School of Mind and Brain, Humboldt Universität zu Berlin, 10117 Berlin, Germany; 4 Einstein Center for Neurosciences, 10117 Berlin, Germany

**Keywords:** Health sciences, Neuroscience, Systems and Computational Biology

## Abstract

The linguistic relativity hypothesis suggests that the way we perceive the world is shaped by the language we speak. Evidence comes from color perception, where Russian speakers, whose language distinguishes between light and dark blue (“goluboj/sinij”), show enhanced discrimination performance for these shades compared to English speakers, who typically use a single term (“blue”) for both. To neuromechanistically explain this phenomenon, we built a brain-constrained neural network simulating neural activity in frontotemporal-occipital cortices. When modeling English speakers’ brains, representational similarity analysis revealed similar activity for different shades of blue carrying the same verbal label. However, in virtual Russian speakers, the same colors carrying different labels induced distinct neural activations. These differences arose from microstructural neural changes, involving shifts in shared and unique neurons encoding color representations. Functionally distinct color representations before labeling were modulated by label learning, thereby facilitating or hindering discrimination. The model also reproduced neurophysiological evidence, supporting its validity. Together, these findings bridge theoretical, linguistic, cognitive, and neuroscientific accounts of how language modulates perception.

## Highlights

• A brain-constrained neural model simulates English and Russian color naming systems
• Shared verbal labels (English) lead to overlapping neural representations of blue shades
• Distinct labels (Russian) enhance neural separation between similar colors
• Label learning induces microstructural changes in visual and frontotemporal cortices

## Graphical abstract

**Figure:**
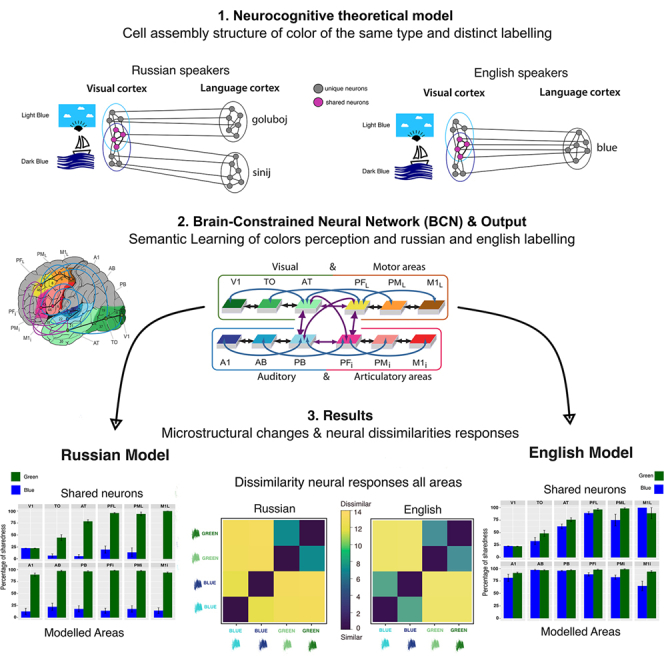

## Introduction

The concept that speakers of different languages perceive the world differently, shaped by the specific lexical and grammatical structures of their languages, has received considerable attention in linguistics, philosophy, and neuroscience. Known as the “linguistic relativity” or “Sapir-Whorf hypothesis,”^1^^,^^2^^,^^3^ this theory suggests that language profoundly influences our cognitive processes. With language woven into nearly every aspect of daily life, from communication to inner speech and thought, it might seem intuitive that language shapes perception. However, are there firm proofs for a causal influence of language on perception? And if so, to what extent does language influence the way we perceive reality and the world around us? In recent decades, scholars have proposed various answers and explanations,^4^^,^^5^^,^^6^^,^^7^^,^^8^^,^^9^^,^^10^^,^^11^^,^^12^^,^^13^ while others have criticized or even dismissed the Whorfian linguistic relativity hypothesis,^14^^,^^15^ so that the debate is still ongoing.

One prominent testing ground for investigating the influence of language on perception is color cognition, that is, how humans perceive and categorize colors. Although color varies continuously along physical dimensions (e.g., hue, brightness, and saturation), speakers of different languages divide this continuous spectrum into distinct categories according to the words available in their color-naming systems. From a universalist perspective, most prominently articulated in the seminal work by Berlin and Kay,^16^ basic color categories are argued to be universal across languages, typically ranging from 2 to 11 fundamental types. According to this view, cross-linguistic differences are considered subtle and tend to be manifested more at higher cognitive tasks than in early perceptual processes (for a review see^17^). In contrast, a range of studies have shown cross-linguistic variation in color perception and categorization among speakers of languages with different color lexicons.^10^^,^^18^^,^^19^^,^^20^ For instance, Russian speakers, who have distinct color terms for light and dark blue, “goluboj” and “sinij,” appear to better discriminate between these shades than they do between different variants of either light or dark blue. A similar category effect could not be found in speakers of English or other languages, where only one frequently used color term is available for the entire category of “blues.”^20^ Similar language-specific category effects have been reported for other color contrasts in Korean^21^ and Berinmo^22^ compared to English, and for bilingual speakers of Lithuanian and Norwegian,^23^ who show enhanced discrimination precisely at those boundaries that are lexically encoded in their language. These results sit comfortably with the idea that the availability of linguistic symbols for different colors enhances the ability of humans to discriminate between them. However, other studies have failed to replicate these effects consistently or reported more nuanced results, suggesting that the influence of language on color discrimination may be more limited and context-dependent than previously assumed^24^ (see also^25^). Beyond behavioral discrimination tasks, converging neurophysiological evidence across speakers of different languages further supports an effect of language on color perception.^4^^,^^10^^,^^19^^,^^26^^,^^27^^,^^28^ One such study examined the visual mismatch negativity (vMMN), a well-known brain index of change detection, in Greek and English speakers.^19^ Similar to Russian, Greek also has distinct names for the two shades of blue. The study found that Greek speakers exhibited pronounced vMMN responses to unexpected changes between the two shades of blue falling under the two different labels. In contrast, English speakers exhibited smaller vMMN responses to shades of blue. Similarly, changes between shades of green, included as control conditions, evoked comparably small brain responses in both Greek and English speakers. As a prominent brain index of change detection is enhanced for perceptual changes marked by different symbols, these results seemingly provide further support for the influence of language on perceptual discrimination ability. A great number of fine studies provide further support for the Whorfian effects in color perception,^5^^,^^8^^,^^10^^,^^26^^,^^29^^,^^30^^,^^31^ as well as in perceiving motion events or space^13^^,^^32^^,^^33^ and whole objects^27^^,^^28^^,^^34^ or tactile stimuli,^35^^,^^36^^,^^37^ along with memory and abstract concept formation.^38^ Still, this leaves open the why- and how-questions about the neural mechanisms behind any Whorfian effect.

One promising approach to address the why- and how-questions is through computational modeling, which provides a unique opportunity to examine the formation of neural circuits and their language-induced changes at multiple levels, ranging from cellular function to neural interaction across large neural ensembles to the level of cortical areas and larger brain parts. Importantly, however, it is essential to develop neural models that closely mimic the intricate neural and structural properties of the human brain in order to gain clues about higher cognitive functions.^39^^,^^40^^,^^41^^,^^42^^,^^43^ In this regard, brain-constrained neural models (BCNs) aiming to offer a neuromechanistic explanation of various linguistic phenomena have been previously developed, mimicking the structural and functional properties of frontotemporal and occipital cortices.^44^^,^^45^^,^^46^^,^^47^^,^^48^^,^^49^^,^^50^^,^^51^^,^^52^^,^^53^ Such previous simulations showed that, in a network with multi-level structural and functional similarity to the human brain, simulation of language learning led to the co-activation of multiple neurons scattered across different cortical regions, which, as a consequence of their regular co-activation, formed strongly interlinked neuronal circuits similar to the cell assemblies postulated by Hebb.^54^ These assemblies provide candidate mechanisms for the neural basis of perceptions, symbols and words in the human brain.^55^^,^^56^^,^^57^

The neural basis of color vision has been studied extensively, and important facts about the contribution of individual cells and their links and interactions are available, which can guide neural modeling. Whereas in the retina and the thalamus of the visual pathway, there are cells maximally responsive to red, green and blue, most cells in the primary visual cortex – the so-called “simple cells” – respond optimally to a given color if it appears against a background of its complementary color (green for red, yellow for blue and vice versa).^58^ In the same way, visual cortex neurons insensitive to color respond maximally to a white spot on a black background or vice versa. In the color-sensitive patches of posterior inferior temporal cortex (the so-called “globs”), there are color-tuned neurons with sensitivity to a wide range of color shades, which are clustered into columns of cells with similar color specificity.^59^ One can conceive cortical wiring patterns that compute sensitivity to various color shades from the output of the aforementioned “simple cells;” for example, a neuron receiving additive input from one red- and one blue-sensitive simple cell would be tuned to magenta, one receiving input from a blue- and a white-sensitive cell would be maximally responsive to light blue. We did not attempt to model this wiring in detail but assumed that there are single neurons specifically responding to different shades of blue, red, green, and so on. In this regard, each color stimulus (e.g., dark blue) would activate a range of such cells, whereas a slightly different color stimulus (e.g., light blue) would activate a distinct but partially overlapping set of neurons. Thereby, individual color “features” were represented by neurons with a narrow tuning to one specific shade, and perceptual similarity was thus modeled as the degree of feature sharing, or, neurally, the overlap of feature neurons, rather than being directly determined by colorimetry parameters. Note that the assumption that similar stimuli activate overlapping sets of neurons, each responding to a specific feature or feature combination of one stimulus or both, is a common and well-founded one in research on visual and other sensory systems.^58^^,^^60^

**Figure 1:**
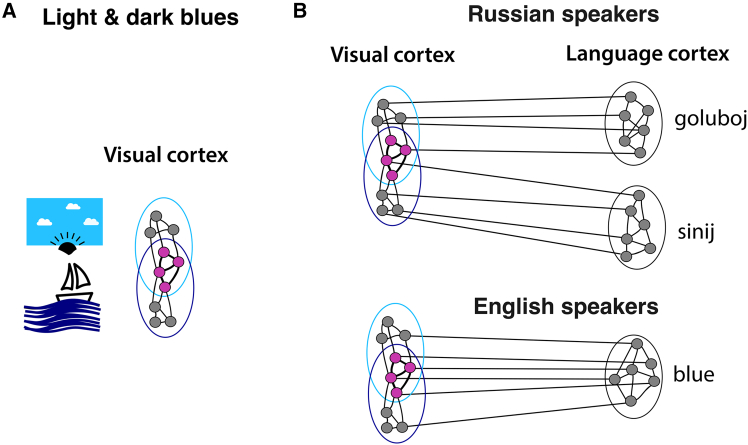
Cell assembly structure of two shades of the same color and the impact of labeling (A) Illustrates the neural assembly structure for two shades of blue (e.g., the light blue of the sky and the dark blue of the sea). Overlapping neural representations (magenta neurons) capture shared color-related features, such as “hue,” while unique neurons (gray neurons) represent features distinct to each specific shade, highlighting the neural encoding of individual color characteristics. (B) Within the context of Russian blue labelling, associating each color shade with a distinct verbal label leads to reduced neuronal overlap by engaging distinct label-related neural circuits a mechanism called “overlap reduction.” In contrast, in English blue labelling, where multiple blue shades are typically grouped into one single label, results in increased neural overlap due to the association of a shared label-related neural circuit.^57^

However, extensive neural overlap between similar concepts or colors can complicate functional separation, as they may activate one another. To address this, it has been suggested that overlap between visual-perceptual neuronal ensembles can be reduced by associating each shade with distinct neural circuits that process symbols or linguistic signs, which are distributed across language-related cortices, thereby increasing the proportion of unique neurons and facilitating functional separation between shades.^57^ Conversely, neural overlap between two shade circuits increases when the same verbal label is associated with two similar shades, thereby further hindering functional separation (see Figure 1). Although this theoretical neural explanation may appear plausible, it requires empirical confirmation. To demonstrate the feasibility of the envisaged mechanism and its consistency with neurobiological reality, it is possible to use biologically founded and mathematically precise neural network models and examine neural circuit formation for color perception and the causal effects of how the acquisition of distinct labels influences these circuits in the context of color stimuli. Of great interest would be models of the brains of speakers of different languages, where the color space is partitioned in different ways by the most frequent color terms, as is the case for Russian and English with regard to shades of blue.

A recent simulation work^48^ applying BCNs has already offered insights into the impact of labeling on neural representations of similar concepts. Specifically, the study investigated the learning of general category terms for several similar objects and specific terms, proper names, each denoting just one object exemplar. The study revealed the formation of neural circuits spread across multiple regions of the network but with distinct neural patterns: some neurons were “shared” across instances, responding to the general category features common to all similar objects, while “unique” neurons selectively responded to features specific to each instance. Interestingly, learning a category name (e.g., “animal” for different dog instances) increased the number of shared neurons activated across all category exemplars, while reducing the number of “unique” instance-specific neurons substantially. In contrast, learning proper names (unique names for each instance) limited the growth of shared neurons and prevented the radical loss of unique neurons observed with category terms. Although this recent simulation study provides insights into how labeling impacts conceptual representation, object perception, and category formation, it did not specifically address the influence of language on what is visually perceived by a language user. Furthermore, as their study targeted category terms and proper names, several instances falling under the same category term were simulated. In the specific case of linguistic relativity, a central question is whether a continuous perceptual gradient is perceived as a single category or split into two distinct categories, depending on language-specific labels. Therefore, a neural model exposed to just 2 partially overlapping cell assemblies appears most appropriate or suitable for investigating the issue. Moreover, in the domain of color perception and discrimination among speakers, it is essential to construct models that simulate speakers with varying color naming systems, such as those of English and Russian speakers. Examining the neural representation of color prior to linguistic labeling and comparing it with the neural patterns that reflect key features of color labeling among English and Russian speakers would offer valuable insights into the neural changes and the impact of language. This comparative strategy has the potential to provide a neuromechanistic explanation of previous evidence on color perception among different speakers, as described above.

To this end, we employed a brain-constrained neural model (BCN) that simulates 12 cortical regions across the frontotemporal occipital cortices to study key features of color perception and the effects of different labeling systems. Specifically, the study proceeded in three simulation steps: First, a BCN model was constructed to simulate color perceptions of two shades of blue and green, along with the phonological formation of words, mimicking a child encountering those colors in the environment and babbling various phonological words in separate instances. Second, the pre-exposed perceptual model was duplicated to create two versions, each used to explore the effects of distinct labeling systems (English and Russian) on the color perception of blues and greens. In the English model, both color shades (blues and greens) shared a single label, while the Russian model applied two distinct labels to the shades of blue, and a single label was used for both shades of green. Finally, in the third step, we simulated color recognition following the semantic learning phase in both English and Russian models to examine differences in their neural responses, comparing the outcomes to a previous EEG study on color perception.^19^ To this end, we asked: (i) whether distinct labeling of two similar shades of color results in differentiated internal neural representations within the modeled regions, (ii) whether attaching one or two distinct labels leads to neural changes in previously formed color representations, and (iii) whether neural activation patterns in the model exhibit similarities to those observed in real brains.

## Results

### General neural network structure and function

**Figure 2:**
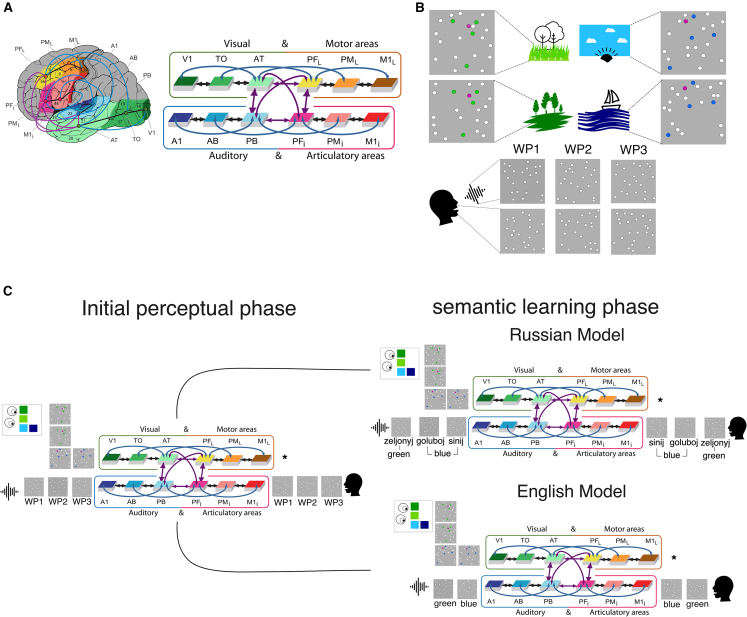
Brain and schematic illustrations of the brain-constrained neural network, including the neural patterns used for stimulation and learning procedure (A) illustrates the structural organization and connectivity of the 12 frontal, temporal, and occipital cortical areas relevant for word meaning acquisition, depicted in the brain and the overall model schematic with corresponding colors of the brain areas. The perisylvian cortex encompasses the inferior-frontal articulatory-phonological system (by bluish and reddish colors), whereas the extrasylvian areas consist of the lateral dorsal hand-motor system (in yellow to brown) and the visual “what” stream responsible for object processing (greenish colors). The numbers indicate the corresponding Brodmann areas (BAs), and the arrows (in black, purple, and blue) represent the long-distance cortico-cortical connections as established in neuroanatomical studies (Figure taken from^51^). (B) Sensorimotor patterns used as inputs for learning depict the overlapping structure created for two shades of blue and green, reflecting shared color features as well as those unique to each color, along with the different neural structural input patterns of the three-word patterns (WPs). (C) Illustrates the initial exposure phase (left panel) and the associative semantic learning procedure (right panel) for the Russian and English models. In the initial exposure model, each color pattern was given as input to V1 mimicking color perception, and the pairs of patterns of the WPs were given as input to A1 and M1i to simulate key aspects of spoken word form (label) processing. The color and word form patterns were presented separately in different learning episodes. The associative semantic learning process involved the simultaneous stimulation of A1 and M1i with the learned auditory and articulatory neural patterns, alongside V1 stimulations with the color patterns that mimic label color association. Note that the Russian and English models differ only in their label assignments: the Russian model assigns distinct labels to light and dark blues (goluboj and sinij) but uses an identical label for shades of green (zeljonyj), whereas in the English model, each pair of color shades shares one label; that is, both shades of blue and green are associated with a single word form pattern.

To simulate color perception and the impact of different labeling, we implemented a Brain-constrained neural network (Figure 2A) comprising 12 cortical frontotemporal and occipital regions relevant for language, visual, and motor processing. At the microstructural level, the model is composed of spiking neurons (integrate-and-fire neurons) that approximate the function of pyramidal neurons in the cortex,^61^^,^^62^ along with inhibitory cells (i-cells) that capture the average activity of local pools of interneurons. The excitatory cells (e-cells) incorporated several properties of biological neurons, summarized as follows.

i. Temporal summation of excitatory and inhibitory inputs,
ii. “All-or-nothing” threshold-based spiking, coupled with neuronal adaptation based on a cell’s recent firing-rate activity.^61^^,^^62^
iii. Incorporation of the white noise process to reflect the spontaneous firing of pyramidal neurons, or the so-called baseline noise.^63^
iv. Synaptic weights undergo modification according to the Hebb-type learning rule, including both long-term potentiation (LTP) and long-term depression (LTD),^54^^,^^64^ as extensively supported by empirical studies.^65^^,^^66^^,^^67^

At the macrostructural level, the spiking neural network mimicked 12 cortical areas of the frontotemporal and occipital cortices, relevant for language processing,^68^^,^^69^^,^^70^ visual perception, and action processing.^71^^,^^72^^,^^73^ Each area was composed of 25 x 25 = 625 e-cells and an equal number of i-cells, with the following implementations.

i. Six areas essential for processing spoken words, located in the perisylvian language cortex, are divided into two distinct modality-preferential systems: The articulatory system, including regions in the inferior face-motor (M1i), premotor (PMi), and prefrontal (PFi) areas, and the auditory system encompasses the superior-temporal primary (A1), belt (AB), and parabelt (PB) areas (Figure 2A highlighted in red and blue).
ii. Six areas outside the perisylvian cortex, involved in visual and action information processing, divided into two processing streams: the ventral visual stream, including primary visual (V1), temporo-occipital (TO) and anterior temporal lobe (AT), and the dorsolateral motor stream, including lateral primary motor (M1L), premotor (PML), and prefrontal (PFL) region (Figure 2A highlighted in green and brown/yellow).
iii. The connectivity structure linking the different cortical regions, based on neuroanatomical evidence, included between-area connectivity (black arrows), long-distance cortico-cortical connections (purple arrows), and non-adjacent jumping links (blue arrows). This connectivity structure was motivated by previous studies using diffusion tensor and weighted imaging (DTI/DWI) in humans and non-human primates (for a full reference, see Table S1 and Tomasello et al. 2018^51^)
iv. The model also included global and local regulation mechanisms (global and local inhibition)^74^^,^^75^ relevant for regulating the local activity of neighboring neurons and across cortical areas during information processing.^76^^,^^77^

The biological principles implemented in the neural network at the micro and macro levels have been proposed to be relevant for simulating higher cognitive functions, such as language and semantic processing, and may be applied to achieve a neuromechanistic explanation of specific observations in language processing, including language-perception interplay.^41^^,^^78^ The properties of individual neurons, the rules governing synaptic plasticity, and the structure of the single-area model are described in more detail in the STAR Methods sections and previous publications,^44^^,^^49^^,^^51^^,^^79^^,^^80^ and have also recently implemented in the NEST simulation framework.^81^

To simulate color perception and assess the impact of distinct labeling on neural representations, we implemented a two-phase learning process (see Figure 2C) based on prior simulation work on rapid symbolic learning^44^ (see also^43^ for discussion on the two learning phases for symbol acquisition). We created 16 networks with different initializations to represent 16 brains of different language learners.

In the first initial perceptual phase, we simulated perceptual and sensorimotor learning for two distinct shades of blue and green, alongside separate episodes of phonological word form learning. Sensorimotor patterns (Figure 2B) were delivered to the respective primary modality regions (color perception to V1; phonological forms to A1 and M1i). This setup simulates a developmental scenario in which a child is first exposed to color referents (e.g., light blue skies, dark blue seas, and various natural greens) in isolated learning episodes, while separately engaging in babbling, producing meaningless phonological forms before any word-referent associations are established (see Figure 2C left panel). Such a pre-exposure phase has been shown to be critical for rapid word-meaning mapping and to improve learning rate in the formation of neural circuits in computational models^44^ (see for a review^43^). Importantly, this initial phase allowed us to examine the formation of color-related neural circuits for similar and distinct color shades prior to any labeling, providing a baseline for examining whether and how subsequent linguistic labeling modulates these pre-formed perceptual representations.

In the second, semantic learning phase, symbolic learning was simulated by co-activating the pre-formed neural representations for colors and word forms, thereby modeling a developmental stage in which a child perceives a familiar color while uttering the corresponding word (see Figure 2C right panel). This stage builds upon the initial perceptual phase: because color- and word-related circuits were initially formed independently, any subsequent changes in their connectivity or internal structure can be directly attributed to the effects of linguistic labeling rather than to perceptual or phonological learning itself. To model language-specific effects, two parallel neural networks were duplicated from the pre-linguistic model obtained in the first phase and subsequently trained to represent English and Russian speakers. In the English model, both shades of blue and green were associated with a single common label for each color, whereas in the Russian model, two distinct labels were assigned to the blue shades, while greens retained a single shared label. This procedure allowed a controlled examination of how distinct labeling systems shape neural representations, both within and across language-specific networks, by directly comparing the pre-labelling (perceptual) and post-labelling (semantic) phases. A detailed description of the learning implementation is provided in the “simulated learning procedures” section of the STAR Methods.

### Representational similarity analysis results

**Figure 3:**
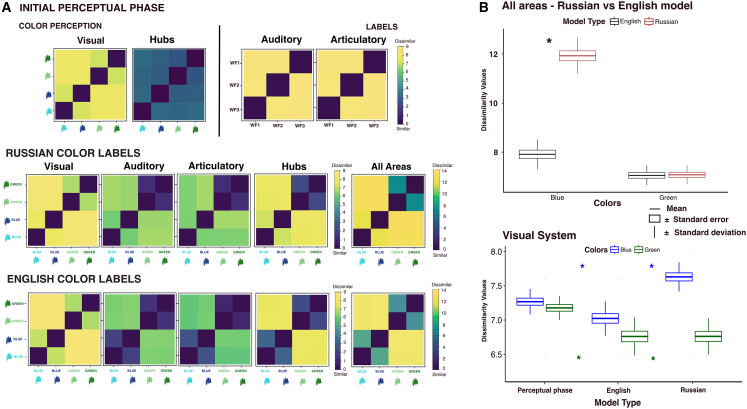
Results of representational dissimilarity matrix (RDMs) of neural activity elicited by color stimuli in the brain-constrained network models Results are shown after perceptual learning and after learning Russian and English color terms. (A) The upper panel shows the Representational Dissimilarity Matrix (RDMs) for the perceptual model, the middle panel for the Russian model, and the bottom panel for the English model. Each RDM is a 4x4 matrix, where each square represents, through color coding, the dissimilarity between the respective pairs of the four color patterns. The dissimilarity measure used was the Euclidean distance, with 0 (dark blue) indicating identical representations. (B) Illustrates the dissimilarity values between blue and green colors across the respective model types, represented by boxplots. These boxplots indicate the mean dissimilarity values (horizontal line), standard error (box), and standard deviation (vertical line). The upper section displays results for all regions, comparing the English (black boxplot) and Russian models (red boxplot) for blue and green colors. Note the significantly higher mean dissimilarity value for blue in the Russian model. The lower panel shows data specific to the visual system (V1, TO, and AT combined), comparing values from the perceptual learning phase to the English and Russian dissimilarity values for the two-color types. A significant decrease in dissimilarity values was observed when a single label was shared between similar shades (blues and greens) compared to the color perceptual phase, with a notably higher dissimilarity value for blue in the Russian model. Asterisks indicate significant differences (Bonferroni comparisons). Differences between blue and green values within each model type were not analyzed, as the input patterns for both color shades were constructed using distinct sets of neurons. Consequently, any observed differences might reflect structural differences in the inputs (e.g., lower green values compared to blue during the perceptual phase).

Following perceptual and sensorimotor (phase1) and semantic (phase 2) learning, the model showed the emergence of strongly connected neurons, the so-called “cell assemblies,”^54^ scattered across the different modeled regions. To examine the impact of labeling on shades of blue- and green-related neural representation, we assessed the dissimilarities (Euclidean distance) in the model’s internal neural representations after semantic learning across different model types. Figure 3 presents the Representational Dissimilarity Matrices (RDMs) for the various modality systems (visual, auditory, articulatory), central hub areas, and data collapsed across 10 relevant regions, illustrating the degree of difference between the corresponding neural representations.^82^ Note that the motor cortices (PML and M1L) were not included, as these regions showed minimal neural activation due to the presentation of uncorrelated input patterns rather than correlated ones to these areas (see STAR Methods section). For completeness, RDM plots for the three motor cortices are provided in the supplementary material (Figure S1). Distinct patterns emerged based on whether identical or different labels were assigned to two shades of the same color. In the Russian model, a pronounced similarity (low dissimilarity value) was observed between the two shades of green that shared the same label, whereas the two blue shades assigned different labels exhibited pronounced dissimilarity (high dissimilarity value) after the acquisition of the corresponding color terms. In the English model, both shades of blue and green, each labeled identically, show higher similarities (low dissimilarity values), which are comparable to those of the green shades in both models (Figures 3A and 3B). These patterns are consistent across different systems, in both the aggregated data from all hub areas and across the relevant regions.

Changes in dissimilarity values were also evident in the visual system when comparing the results from the initial perceptual learning and post-symbolic learning. Shades of the same color receiving identical labels (green for Russian and green and blue for English models) showed lower dissimilarity values as compared to the initial exposure in the perceptual learning phase. In contrast, shades of color receiving two distinct labels (blues in the Russian model) showed higher dissimilarity values after color term learning (Figure 3B lower panel).

#### Russian vs. English models

The visual observations described above were confirmed by repeated-measures 2X2 ANOVAs (Model Type X Color) conducted across all relevant regions. The analysis revealed significant main effects of Model Type (F_1,12_ = 158.9, *p* = 0.001) and Color (F_1,12_ = 326.4, *p* < 0.001), along with a significant interaction between these factors (F_1,12_ = 213.3, *p* < 0.001). Further examination through pairwise post hoc tests indicated that the significant interaction was primarily due to the differences in dissimilarity scores for the blue shades between the English (mean = 7.92) and Russian (mean = 11.93) models (*p* < 0.001, 95% CI = [-4.64, −3.39]). In contrast, the dissimilarity scores for the green shades showed no significant differences between English (mean = 7.05) and Russian (mean = 7.08) models (*p* = 0.788, 95% CI = [-0.21, 0.16]; refer to Figure 3B upper panel). This indicates that the dissimilarity values between the two models differed in the shades of blue, with the Russian model displaying higher dissimilarity values and the English model showing lower ones.

The 2x2 ANOVA conducted on the combined data from all hub regions showed consistent results with those obtained from the analysis on all regions described above: A main effect of Model Type (F_1,12_ = 193.07, *p* < 0.001) and Color (F_1,12_ = 190.31, *p* < 0.001), along with a significant interaction (F_1,12_ = 201.88, *p* < 0.001). Also, here the pairwise post hoc tests revealed that the significant interaction was primarily due to the differences in dissimilarity scores for the blue shades between the English (mean = 3.26) and Russian (mean = 7.17) models (*p* < 0.001, 95% CI = [-4.44, −3.38]), while no significant difference was found for the green shades between the two English (mean = 2.27) and Russian (mean = 2.23) models (*p* = 0.773, 95% CI = [-0.25, 0.32]).

Two additional 2x2 ANOVAs (Learning Phase X Color) were conducted by comparing each model type (English and Russian) with the perceptual model on the data extracted from the model’s visual system following color stimulation after perceptual and after semantic learning phases (see Figure 3B lower panel).

#### Russian vs. perceptual model

The Russian network exhibited a main effect of Color (F_1,12_ = 34.6, *p* < 0.001) and a significant interaction between Learning Phase and Color (F_1,12_ = 86.3, *p* < 0.001). The subsequent pairwise post-hoc tests indicated a significant increase in dissimilarity values (*p* < 0.001, 95% CI = [-0.48, −0.23]) for the shades of blue for Russian model (mean = 7.62) as compared to the initial perceptual model (mean = 7.27), and a marked reduction in dissimilarity values for the shades of green (*p* < 0.001, 95% CI = [0.30, 0.54]) when comparing the Russian (mean = 6.75) model versus the initial perceptual (mean = 7.18) model. These findings confirm that labeling affects preformed representations in the visual system: distinct labels for blue shades enhanced representational dissimilarity, whereas identical labels for green shades diminished it.

#### English vs. perceptual model

The English network showed only a main effect of Learning phase (F_1,12_ = 33.38, *p* < 0.001) without a corresponding significant interaction between Learning phase and Color (F_1,12_ = 2.28, *p* < 0.157). Post-hoc comparisons exhibited a decrease in dissimilarity values for both blue (*p* = 0.03, 95% CI = [0.03, 0.46]) and green (*p* < 0.001, 95% CI = [0.29, 0.55]) shades when comparing the English model (blue mean = 7.02, green mean = 6.76) versus the initial color perceptual model (blue mean = 7.27, green mean = 7.18), further supporting the increased neural similarity resulting from identical labeling.

These statistical results were consistent with results obtained from analyses of the visual AT hub region across learning phases (see supplemental information for more details).

### Microstructural changes: Neuron type (shared vs. unique) results

**Figure 4:**
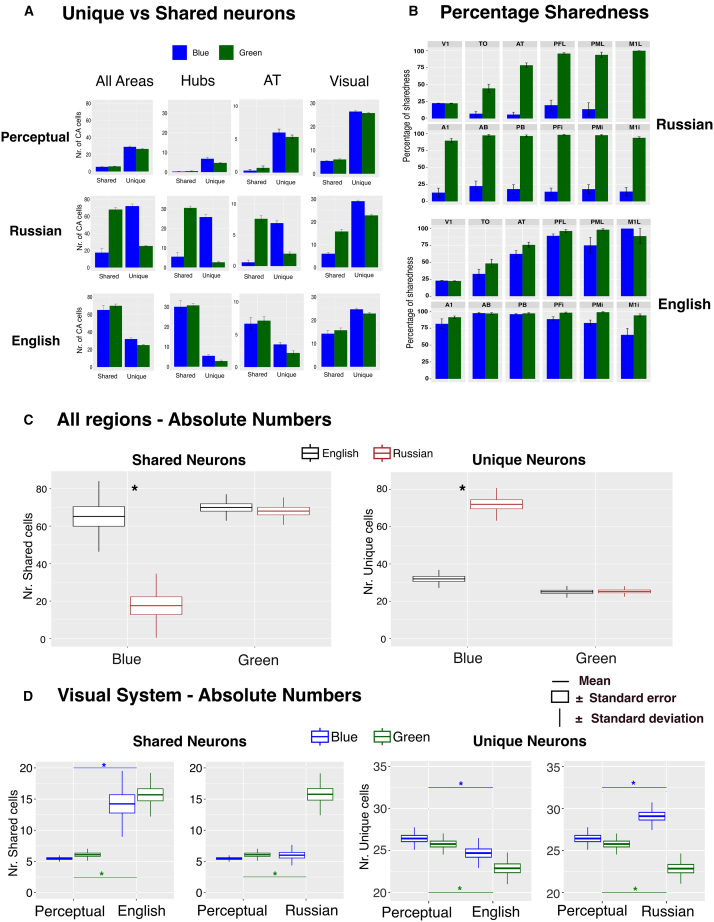
Number of neuron types involved in color processing across the different model types (A) Bar graphs illustrating the number of unique and shared neurons between the two shades of the same color (blue and green) across all relevant areas, all hubs, the AT region, and the visual system for the pre-label (perceptual) (top panel), Russian (mid panel), and English models (bottom panel). (B) Bar graphs of the percentage of shared neurons between blue and green shades in each modeled region for the Russian (top panel) and English (bottom panel) models. Note that the percentage of neurons in the stimulated visual area (V1) is similar between models due to the predefined stimulation pattern. Error bars represent standard error. (C) Boxplots compare the number of shared (left panel) and unique (right panel) neurons for blue and green across all relevant regions in the English and Russian models. (D) Boxplots compare shared (left panel) and unique (right panel) neurons for the two color categories in the visual system, contrasting each trained model with the initial pre-label (perceptual) model. In all plots, the horizontal line denotes the mean, the box the standard error, and the vertical lines the standard deviation. Asterisks indicate significant differences (Bonferroni comparisons).

To further examine the impact of color labeling within and between the English and Russian models, as well as in comparison to the initial perceptual learning phase, we quantified the density of shared and unique neurons among the neural representations of the color shades (blue and green, for more details on the calculation see Methods section). Visual observation of the distribution of neuron type sizes, influenced by whether distinct or identical labels were associated with two shades of color, revealed a consistent pattern: In the Russian model, associating distinct labels with different blue shades significantly increased the number of unique neurons. Conversely, associating identical labels with two shades of the same color (blue and green in the English model, and green in the Russian model) led to a substantial increase in the density of shared neurons. This pattern persisted across all relevant regions, including the central hubs and the visual AT region, as illustrated in Figure 4A.

To further analyze differences in neuron type across models, we depicted the percentage of shared neurons or “sharedness” [Number of Shared Neurons/(Number of Shared Neurons + Number of Unique Neurons) ∗ 100], per model area, averaged across neural representations and models (see Figure 4B). Notably, in the Russian model, shared neurons for blue shades labeled differently were consistently below 25% across modeled regions – a partial consequence of the overlap between stimulation patterns. In contrast, green shades receiving identical labels showed a higher sharedness percentage, ranging from 30% to over 50% in the visual and hub regions and almost 100% in the perisylvian language regions (from A1 to M1i). Similarly, the English model, employing identical labels for blue and green shades, demonstrated high sharedness percentages similar to those for greens in the Russian model. High sharedness between the CAs activated by two perceptual patterns was thus due to the availability of common labels.

When comparing visual system data before and after symbolic learning, distinct patterns emerged: assigning different labels to two shades of the same color (blue in the Russian model) led to an increase in unique neurons, whereas the count of shared neurons remained unchanged relative to the perceptual learning model. This stability in shared neurons, as well as their generally low number, might be due to the perceptual input patterns to V1, which had only a few shared neurons in the first place. Conversely, assigning identical labels to two different shades (green in the Russian model, and both blue and green in the English model) resulted in an increase in shared neurons and a decrease in unique neurons, as illustrated in Figure 4D.

#### Russian vs. English model

The above visual observations were confirmed by the 2 × 2 × 2 repeated measures ANOVA (Model Type X Color X Neuron Type) on the data from all relevant regions. The statistical results showed a main effect of Model Type (F_1,12_ = 7.34, *p* = 0.019) and Neuron Type (F_1,12_ = 44.98, *p* < 0.001) and a three-way significant interaction between Model Type, Color, and Neuron Type (F_1,12_ = 309.44, *p* < 0.001). To disentangle this three-way interaction, further statistical analyses were conducted for each Model Type separately (within-model analysis). For each of them, a 2x2 ANOVA (Color x Neuron Type) was performed on the data.

In the Russian model, a significant Color × Neuron Type interaction (F_1,12_ = 178.83, *p* < 0.001) was revealed. Pairwise comparison indicated more unique than shared neurons for blue (unique mean = 72.04, shared mean = 17.54, *p* < 0.001, 95% CI = [-67.58, −41.42]) and more shared than unique neurons for green (shared mean = 68.00, unique mean = 25.27, *p* < 0.001, 95% CI = [37.25, 48.21]).

In contrast, this significant interaction was not observed in the English model (F_1,12_ = 3.14, *p* = 0.102), indicating a comparable distribution of shared and unique neurons between the blue (shared mean = 65.15, unique mean = 32.00, *p* < 0.001, CI = [20.25, 46.06]) and green (shared mean = 69.92, unique mean = 25.08, *p* < 0.001, CI = [39.95, 49.75]) shades, both of which were identically labeled (see Figure 4C). The English model showed only a main effect of Neuron Type (F_1,12_ = 165.88 *p* < 0.001), indicating more shared neurons than unique ones, regardless of color type.

The 2x2 ANOVA conducted on the combined data from all hub regions confirmed these results; in particular, a significant interaction between Color and Neuron Type was only observed for the Russian model (F_1,12_ = 216.27, *p* < 0.001), but not for the English model (F_1,12_ = 0.85, *p* = 0.375).

A similar significant interaction result was found for the visual AT hub region (see supplemental information).

To compare neuron types between the English and Russian models, 2x2 ANOVAs (Model Type × Color) were conducted separately for unique and shared neurons in all relevant areas, with the following results:

For unique neurons, significant main effects of Model Type (F_1,12_ = 152.76, *p* < 0.001) and Color (F_1,12_ = 288.05, *p* < 0.001) were observed, along with a significant interaction (F_1,12_ = 192.42, *p* < 0.001). Post hoc tests showed significant differences for blue shades (*p* < 0.001, 95% CI = [-46.59, −33.49]) but not for green shades (*p* = 0.761, 95% CI = [-1.54, 1.16]) between the models.

For shared neurons, significant main effects of Model Type (F_1,12_ = 163.10, *p* < 0.001) and Color (F_1,12_ = 21.60, *p* < 0.001) were also found, with a significant interaction (F_1,12_ = 326.75, *p* < 0.001). Post hoc tests again revealed significant differences for blue shades (*p* < 0.001, 95% CI = [41.41, 53.82]) but not for green shades (*p* = 0.257, 95% CI = [-1.60, 5.44]) between the models. These results highlight differences in shared and unique neuron representations for blue but not green shades between the English and Russian models, consistent with the results above.

The results remained consistent when data from all hub regions were analyzed. For unique neurons, there were main effects of Model Type (F_1,12_ = 199.54, *p* < 0.001) and Color (F_1,12_ = 329.04, *p* < 0.001), with a significant interaction (F_1,12_ = 233.46, *p* < 0.001). Post hoc tests showed differences for blue shades (*p* < 0.001, 95% CI = [-23.32, −17.45]) but not green (*p* = 0.579, 95% CI = [-0.54, 0.93]). Similarly, shared neurons showed main effects of Model Type (F_1,12_ = 150.26, *p* < 0.001) and Color (F_1,12_ = 19.92, *p* < 0.001), with a significant interaction (F_1,12_ = 225.49, *p* < 0.001). Post hoc tests again revealed differences for blue shades (*p* < 0.001, 95% CI = [20.66, 28.26]) but not for greens (*p* = 0.776, 95% CI = [-0.99, 1.30]).

Results were consistent with analyses of the AT visual hub region (see supplemental information).

#### Russian vs. perceptual model

The analysis of the visual areas revealed significant main effects of Label (F_1,12_ = 169.68, *p* < 0.001), Color (F_1,12_ = 5.55, *p* < 0.036), as well as for Neuron Type (F_1,12_ = 2070.42, *p* < 0.001), along with a three-way interaction (F_1,12_ = 102.80, *p* < 0.001). Post hoc tests showed an increase in unique neurons for blue shades in the Russian as compared to the Perceptual model (Perceptual mean = 26.42, Russian mean = 29.08, *p* < 0.001, 95% CI = [-3.60, −1.71]) and a decrease in unique neurons for green shades in the Russian versus the Perceptual model (Perceptual mean = 25.77, Russian mean = 22.85, *p* < 0.001, 95% CI = [2.09, 3.76]). Shared neurons showed no difference for blue (Perceptual mean = 5.46, Russian mean = 6.00, *p* = 0.252, 95% CI = [-1.51, 0.44]) but a significant increase for green shades for the Russian as compared to Perceptual model (Perceptual mean = 6.08, Russian mean = 15.77, *p* < 0.001, 95% CI = [-11.44, −7.94]) with identical labeling.

#### English vs. perceptual model

The English model in the visual areas showed significant effects of Label (F_1,12_ = 220.21, *p* < 0.001) and Neuron Type (F_1,12_ = 1466.04, *p* < 0.001), with an interaction between Label and Neuron Type (F_1,12_ = 130.58, *p* < 0.001), but no effect of Color (F_1,12_ = 0.04, *p* = 0.83) or triple interaction (F_1,12_ = 0.57, *p* = 0.464). Shared neurons increased in the English model as compared to the Perceptual model for both blue (Perceptual mean = 5.46, English mean = 14.23, *p* < 0.001, 95% CI = [-11.85, −5.69]) and green (Perceptual mean = 6.08, English mean = 15.69, *p* < 0.001, 95% CI = [-11.49, −7.74]). Meanwhile, unique neurons decreased in the English model as compared to the Perceptual model in both blue (Perceptual mean = 26.42, English mean = 24.69, *p* < 0.028, 95% CI = [0.22, 3.24]) and green (Perceptual mean = 25.77, English mean = 22.88, *p* < 0.001, 95% CI = [1.99, 3.78]) after identical labeling.

These findings were replicated in the visual connector hub region, AT (see supplemental information).

#### Neurophysiological indices of color perception - Results

**Figure 5:**
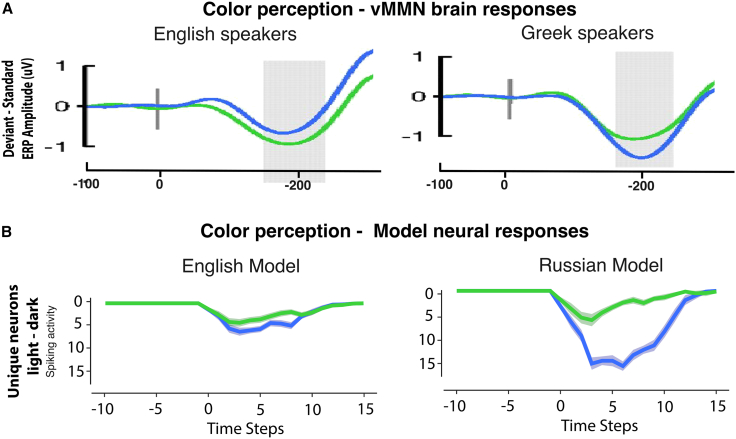
Comparison of real and simulated brain activation in color recognition processes (A) Neurophysiological responses to shades of blue and green in a visual mismatch negativity (vMMN) study (adapted from Thierry et al., 2009^19^). This study examined neural responses to unexpected changes in color by presenting a sequence of repeated “standard” color shades (e.g., dark blue) intermittently interrupted by “deviant” shades (e.g., light blue). The plot compares Greek and English speakers by subtracting the neural response to the standard from the deviant, showing a stronger neural MMN response to blue than green in Greek speakers, an effect not observed in English speakers. This effect is attributed to linguistic differences: Greek employs two distinct labels for shades of blue, while English uses a single label, influencing perceptual categorization. (B) The graphs depict model responses of unique neurons to blue and green in both the English and Russian simulations during color perception following semantic learning (see Methods section). By isolating the activity of unique neurons, we assessed the network’s capacity to distinguish between shades, with the proportion of activation specific to dark or light color shades serving as an indicator of the network’s computational ability to separate these shades from one another. This approach parallels the visual mismatch negativity (vMMN) response to colors, where neural activity reflects the brain’s detection of unexpected changes in color shades. However, stronger vMMN is detected when different color categories result from distinct labeling, thus capturing the activation pertinent to the lexical distinction in the Russian model upon detecting an expected color change. Consistent with the vMMN study, the Russian model shows a stronger neural response of unique neurons of dark/light blue compared to dark/light green, a pattern not evident in the English model.

The analysis of unique neural responses over time, obtained by subtracting shared neurons between light and dark colors from the total activation (see Methods section), revealed distinct response differences over time between the English and Russian models. Figure 5B shows a more pronounced activation for blue compared to green in the Russian model, while responses in the English model appear similar for both colors. The visual observation was statistically confirmed by a 2x2 ANOVA (Model Type x Color) on the unique neural responses from time steps 2–12, which revealed main effects of Model Type (F(1,12) = 54.9, *p* < 0.0001) and Color (F(1,12) = 56.6, *p* < 0.0001), as well as a significant interaction between the two factors (F(1,12) = 64.4, *p* < 0.0001). To further assess the effects of color within each model type, we conducted post-hoc Bonferroni paired t-tests. As predicted, there was a significant effect of Color in the Russian model (*p* < 0.0001, 95% CI = [6.65, 9.61]), but not only a nearly significant difference for the English model (*p* = 0.066, 95% CI = [-0.13, 3.40]).

## Discussion

Simulating color perception and label acquisition in a brain-constrained neural network (BCN) of the human cortex revealed critical insights into the neural mechanisms of how language modulates color perception. When sensorimotor patterns were given as input to the networks’ primary visual system (V1), thought to code key features of similar shades of blue and green colors, the network showed successful color perceptual learning. Likewise, sensorimotor inputs to the primary auditory (A1) and articulatory cortices (M1i), thought to mimic auditory and articulatory signals corresponding to spoken word forms or labels, led to the formation of distributed word-related neural circuits. These processes simulated a child being exposed to different colors and color words before forming associations between the two.

During the semantic learning phase, pairs of learned color and word form patterns were co-presented to the primary model regions (V1, A1, M1i), simulating a typical learning situation in which a word is spoken when the corresponding color is perceived. Crucially, whether each color shade was paired with a single label (as in English “blue”) or two distinct labels (as in Russian “goluboj” for light blue and “sinij” for dark blue) determined how distinct the neural representations for those shades became. In the Russian model, the two blue shades produced markedly dissimilar neural activation patterns due to their distinct labels, whereas in the English model, which used a single label, the representations were notably more similar. Both models, however, showed equally low dissimilarity for green shades, which shared one label across both languages (Figures 3A and 3B). These differences in neural response dissimilarities in the model’s internal perception reflected microstructural neural changes: an increase in shared neurons between similar color shades assigned a single label, along with a decrease in specific unique neurons; conversely, an increase in unique neurons for each shade was noted when distinct labels were assigned (see Figure 4A). Importantly, similar effects and neural microstructural changes were evident in the model’s visual cortices when comparing the color initial perception phase to the symbolic learning phases in both English and Russian models.

Based on these results, two main conclusions can be drawn. (i) Assigning distinct labels to shades of the same color enhances their functional separability of the underlying neuronal assemblies, which means better perceptual discrimination of these shades at the cognitive level. In contrast, associating a single label diminished the proportion of distinct neuronal elements between color shade circuits and increased their overlap, thus resulting in reduced perception discrimination. (ii) Labeling modulated neural representations formed independently of direct language experience, indicating that labeling has an impact on the neural encoding of color. The validity of our simulation is supported by the similarities between simulated neurophysiological responses to color perception and previous neurophysiological findings (Figure 5). Below, we discuss this evidence across previous studies on color perception, the model explanation thereof and previous simulation work.

### Neuromechanistic explanation of the Whorfian effect on color perception

A large body of research on the relativity of color cognition has demonstrated significant differences in color perception and categorization among speakers of different languages.^5^^,^^10^^,^^19^^,^^20^^,^^26^^,^^30^^,^^31^^,^^83^ Studies show that speakers of languages with distinct labels for light and dark blue demonstrate enhanced cross-boundary discrimination between these shades compared to speakers whose languages lack this distinction.^20^^,^^31^^,^^84^^,^^85^ Neuroimaging research has revealed that these perceptual differences led to distinct patterns of neural activity in the human brain across different speakers.^19^^,^^83^^,^^86^^,^^87^^,^^88^^,^^89^ Furthermore, it has been shown that categorical perception of colors is already present in infants prior to language exposure,^90^^,^^91^ raising further questions about why differences in color perception and discrimination exist among speakers with different color naming systems.

Our simulation study provides a neuromechanistic explanation for these behavioral and neurocognitive effects. By employing a brain-constrained neural network with spiking neurons and neuroanatomically structured cortical regions, we demonstrate that color representations were initially distinct and localized in the visual cortex, aligning with evidence for categorical color perception prior to language exposure.^90^^,^^91^ Critically, these color-related neural representations became either more similar or more distinct depending on whether a single label or two labels were applied. This labeling led to the model’s internal perception of either a single category or separate categories, respectively. Yet, which specific neurobiological mechanisms implemented in the model are responsible for driving these changes?

The principles of correlation learning and Hebbian synaptic plasticity (weakening and strengthening of synapses)^54^^,^^64^ enabled the successful mapping between pre-formed neural representations of color and labels across the modeled regions, thus successfully forming symbolic neural representations. Notably, when the same label was shared between two distinct color representations, repeated correlated activity strengthened the connections between each color representation and the shared label. Over time, the label’s neural representation co-activated both color circuits upon the presentation of either shade, increasing their similarity due to shared labeling, and thereby enhancing the extent of shared neural matter. As a result, the model perceives the two shades as belonging to a single category rather than as distinct entities. In contrast, when distinct labels are attached to each color representation, the opposite effect occurs. Each color representation strengthens its connections to the specific neurons of its respective label representation, leading to “overlap reduction.”^57^ This, by definition, increases the number of neurons that receive strong input when each shade of color is activated, simply because activation spreads from the color shade circuit to that of the linked specific symbol (see Figure 1).

Furthermore, the present findings go beyond previous proposals, which show that distinct labeling actively modulated previously formed color neural representation without symbol association. When one label was applied to a pair of similar colors, their neural representations previously formed in the visual system became more similar; when two distinct labels were applied, the representations grew more dissimilar (see Figure 2B lower panel). These effects resulted from either an increase in shared neural material or an increase in unique neural matter, depending on whether one or two labels were paired with each color representation, respectively (see Figures 3A and 3B). These neural microstructural changes are best explained by the same mechanisms of correlation learning. When a single label is applied to two shades of color, instance-specific neurons tend to remain silent and gradually disconnect from shared neurons through long-term depression, a process known as “neurons out of sync delink.”^54^^,^^64^ Conversely, when two distinct labels are applied to similar color shades, shared neurons tend to disconnect, reducing overlap, while instance-specific neurons increase in response to highly correlated activity associated with unique specific symbols. These effects are prominent in the model’s central hub regions, which are less influenced by direct input, as well as in visual cortices and the hub ATL alone. These results are in line with previous fMRI findings in ventral occipital regions (i.e., V4v and VO1), which showed that neural representations of color become categorically structured in alignment with linguistic color categories, suggesting that language-related influences shape color perception at the level of visual cortex.^92^ However, our simulations go a step further by showing how labeling actively modulates color perception. The results suggest that these differences arise not only from the assignment of one or two distinct label-related circuits^57^ but also from labelling-driven changes that result in either overlap expansion^48^ or an increase in the proportion of instance-specific (unique) neurons.^93^ Overall, we provide a neuromechanistic account for both behavioral differences in color discrimination tasks and cross-linguistic differences in neural activation patterns.^20^^,^^31^

The present simulation results extend a recent computational simulation work using a similar brain-constrained model that simulated the learning of general category terms for several similar objects and specific terms, proper names, specific to each object exemplar.^48^ The study observed that category name learning led to an increase in the number of shared neurons activated by all exemplars of the category. Our findings align with these results, showing a similar increase in shared neural matter when a single label was applied to two similar shades of color. However, in the previous simulations, learning a proper name for each exemplar within a category surprisingly led to a slight decrease in exemplar-specific neurons, with no change in shared neural matter. In contrast, our results show that assigning two distinct labels to similar color shades lead to an increase in unique neural matter, thereby leading to more distinct neural circuits for each shade. One explanation for these differences in results is that the previous simulations trained the network with inputs from both primary visual and motor regions, simulating shared sensorimotor features of similar objects.^48^ This setup may have biased the model toward increased shared neural matter in central hub regions, where converging neural activity from both modalities may limit the development of instance-specific neural matter to each instance. In our present simulation, we addressed the broader English “blue” category versus the more specific Russian distinction by providing input only to the visual system, thereby minimizing the impact of input and activity flow in cross-modal convergence or hub regions. Additionally, we directly simulated color perception and semantic learning within the same neural architecture through a two-stage process^43^^,^^44^: initial color perception, followed by symbolic learning. This allowed us to examine the impact of labeling on previously established color representations more directly. In contrast, previous simulation studies modeled category formation separately, using distinct models: one without symbols and two separate models incorporating symbolic learning for categories and proper names. As an additional, independent factor, Nguyen et al. modeled 3 real-world instances of a concept as overlapping cell assemblies [with 50% of CA neurons in specific and overlap parts, respectively] so that each of the shared neurons activated 3 times as often than the unique and specific neurons; in our simulations, this relationship was just 2:1. This difference also contributed to the greater role unique neurons played in the present simulations in separating perceptual representations. In particular, in the Russian simulations, the relatively small percentage of overlap neurons reduced their connection weights to one of the unique color shade neurons whenever the other set of shade-specific neurons was active, thus leading to a degree of isolation of the small set of overlap neurons (see also^93^). Because the overlap set was small [just 22.8% of CA neurons] and isolated, it did not efficiently recruit additional neurons, as the larger sets of unique neurons [77.2%] could, so that the overgrowth of unique neurons in the present Russian model was not observed in Nguyen et al.^48^ simulation work.

Another fine computational work has examined the effect of language on low-level perceptual representations^94^ by using a simple recurrent neural model with three layers representing label, hidden, and perceptual representations. The network successfully learned novel label items, with category activation being stronger when connected to a label compared to a disconnected label condition, in which the label was prevented from influencing the visual perceptual representation. Although this early model provides valuable insights into the role of labeling and category formation, it is limited in offering a neuromechanistic explanation, particularly regarding label-induced microstructural neural changes. Additionally, as noted by the author, the three-layer model does not clarify whether labels are inherently “special.” Here, we demonstrate that labels are indeed special: symbolic learning with one or two distinct labels either diminishes or enhances perceptual color categories, reshaping previously formed color categories that were pre-formed without linguistic experiences. The present results complement other computational neural network studies that have employed diverse architectures and deep-learning methods. For instance, some models have used feedforward networks with three hidden layers to develop color-naming systems resembling human language, based on color discrimination tasks and discrete communication systems.^95^ Others have explored the emergence of color categories during visual skill acquisition using convolutional neural networks trained on object recognition in natural images.^96^ Additionally, work with pretrained artificial neural networks has suggested that categorical color perception may be partly language-independent, though shaped by linguistic labels during development.^97^ While these approaches have advanced our understanding of how categorical color perception may arise in artificial systems in different tasks and situations, they do not implement biologically grounded neuroanatomical structures or mechanisms. In particular, they lack key biological principles, such as the anatomical organization and connectivity of language and sensorimotor systems and unsupervised plausible synaptic learning rules, which have been defined as relevant to provide a mechanistic explanation of neural processes for higher cognitive functions, such as linguistic phenomena.^39^^,^^40^^,^^41^^,^^42^^,^^43^ The present study incorporates these principles by implementing a large-scale spiking neural network model constrained by the human neuroanatomy of 12 cortical regions involved in language and sensorimotor processing.^44^^,^^49^^,^^51^^,^^52^^,^^56^^,^^63^^,^^93^^,^^98^^,^^99^^,^^100^^,^^101^ Crucially, our two-phase learning model equipped with Hebbian plasticity demonstrates how neural representations of color perception can emerge without linguistic input and how these representations are modulated by language-specific labels. This provides a biologically plausible explanation for well-documented behavioral and neurophysiological differences in color perception across language groups.^10^^,^^19^^,^^20^^,^^30^^,^^83^

### Neurophysiological neural responses to color perception: Model vs. brain responses

The neural semantic circuits formed for color terms in the Russian and English models were reactivated via visual color patterns in the primary visual (V1) region to simulate the cortical temporal dynamics of color perception observed in a previous EEG study^19^ and to validate the model's prediction on language impact on color perception between the two models. Thierry et al.^19^ investigated color perception of blue and green shades in Greek and English speakers using the visual mismatch negativity (vMMN) paradigm, which captures the brain’s automatic response to deviations in visual stimuli from a repeated pattern. Similar to Russian, Greek has distinct frequent symbols for light and dark blue, but not for different shades of green. In this study, standard stimuli involved repeated presentations of a specific color shade (e.g., dark blue), while deviant stimuli, consisting of different shades of the same color (e.g., light blue), occasionally interrupted the sequence. The study reported stronger activation for blue deviants compared to green deviants after a sequence of standard color stimuli in native speakers of Greek. In contrast, in speakers of English, there were no clear differences between the brain responses to greens and blues (see Figure 5A). The authors argue that the observed neurophysiological difference is best explained by language-induced differences in color perception mechanisms.

Our simulation reproduces the experimentally observed stronger activation for blue deviant colors in Russian (Greek) speakers as compared with English speakers or green deviants. These simulations make evident that this physiological difference is due to the additional activation of instance-specific (unique) neurons to the deviant color shade. Note that, when simulating a mismatch negativity experiment, the standard stimulus’s representations must be assumed to be tonically activated by the repeated standards, whereas the rare occasional deviant stimulus leads to the additional activation of its specific, unique neurons. Therefore, the number of newly activated specific (unique) neurons to the deviant stimulus color may serve as a proxy for the MMN responses (see also^102^). Specifically, when standard colors are repeatedly presented, their neural circuits are consistently activated. If the deviant color shares most of its neural representation with the standard color, as observed in English speakers, the activation difference should be reduced. Conversely, when the shared neural representation between standard and deviant color shades is minimal, with a higher proportion of instance-specific neurons for each color shade, the activation difference should be more pronounced. By plotting the unique neural responses during color perception with input given solely in V1 in the two model types, we observed similarities in activation with the previous study.^19^ Specifically, when computing the differences in instance-specific unique neural activation between blue and green shades in the Russian and English models, we found stronger activation for blue compared to green shades in the Russian model, whereas this difference was less pronounced in the English model. This demonstrates how the modeling approach can replicate and mechanistically explain differences in categorical color perception observed at the neurophysiological level, such as variations in MMN responses.

### Limitations and future directions

In this simulation, we modeled key aspects of color perception, specifically the similarities between colors encoded by shared and unique neural features. However, actual inputs to the brain, especially in color perception, are far more complex. Specialized neurons in the visual cortex respond to specific wavelengths, contrasts, and hues, features not implemented in our model’s visual system.^103^^,^^104^^,^^105^^,^^106^^,^^107^ While our study primarily examined the effects of labeling and the differences in color naming systems between English and Russian speakers on color neural representations, future research should strive to replicate this simulation with more realistic input patterns to better capture more realistic neural color encoding in the visual system. In particular, fine-grained similarities between closely related shades (e.g., light blue vs. light green or darker variants) could be examined in relation to distinct labeling systems, especially once more realistic encoding schemes are implemented.

Moreover, previous studies have used a broader range of shades of the same colors with gradual differences between them.^20^ A possible next step would be to follow this approach by designing a more complex set of color stimuli and varying the degree of overlap. For instance, implementing sensorimotor input patterns by gradually increasing the overlap of neural matter between shades of the same color would allow us to examine whether this increased overlap still results in functional separation before language exposure and whether labeling might help to make them functionally separable or not. Consider distinguishing between two very similar colors (two light blues or think about twin brothers), as often, language alone is insufficient to make them easily distinguishable. Hence, future research should investigate the limits of language’s impact on making very similar perceptual entities functionally separable in the human brain. This could help us understand the boundaries of language’s role in differentiating closely related perceptual experiences. In the same vein, simulating the acquisition of multiple color categories (e.g., yellow, red, and orange) and their shared feature structures would further elucidate how interactions among different color categories shape their neural representations. Additionally, in typical learning scenarios, colors and words are often not presented simultaneously. Frequently, the word is heard first, followed by the color, or vice versa, with a short time delay between presentations. Future studies should closely examine the effect of such temporal variability to better understand how these temporal differences may influence neural representations and the categorization processes.

Finally, the use of color terms by specific language communities, where certain shades are preferentially associated with particular referents (e.g., Russian speakers describing light-blue eyes with “goluboj” and blue whales with “sinij”), may also influence microstructural changes in shared and unique neural subpopulations due to distinct co-occurrence patterns in real-world usage. These differences could be further quantified through corpus-based analyses of color-term frequencies, collocations, and contextual distributions, providing empirical constraints for refining and validating brain-constrained modeling approaches. Along similar lines, how language-specific color categorization shapes communication more broadly, for instance, how color terms are used and interact across different contexts and communicative functions understanding (e.g., requests, naming, questions, and statements; see reviews in^108^^,^^109^), remains largely understudied. Further neuroimaging, behavioral, and computational work is needed to address these questions.

### Conclusion

The aim of the present simulation study was to provide a neural mechanistic explanation of the Whorfian effect, as documented in cross-linguistic behavioral and brain studies on categorical color perception. Specifically, we asked: How do learning one versus two labels for two shades of the same color shape their neural representations in the brain? To address this question, we use brain-constrained deep neural networks to simulate the brain processes of color perception and color word learning, in particular of shades of blue and green, in speakers of Russian (who use two distinct labels for light and dark blue) and English (where there is only one predominant term).

We found substantial neural changes of the perceptual representations activated by similar color shades after linguistic or semantic learning. The neural representations of two color shades became highly similar after learning a single color term for both, due to an increased number of shared neurons across neural circuits. In contrast, learning separate color terms for each shade produced distinct representations, amplifying the number of specific neurons in each circuit. The model shows that language can act as a modulator of color perception, either sharpening or blurring perceptual categories and therefore enhancing or reducing the discrimination of differences between color stimuli. Furthermore, the model was able to simulate previous neurophysiological results about linguistically modulated brain responses to color stimuli recorded with the EEG. Most importantly, the current work not only replicates previous studies but also offers a possible explanation of the neural mechanisms involved. This explanation is based on correlation learning and unsupervised Hebbian synaptic plasticity, which strengthens or weakens connections between neurons depending on whether these neurons were activated together or separately. This study provides a neurobiological account of both the Whorfian effect and the related color perception phenomena, well-documented in the brain and cognitive sciences.

## Resource availability

### Lead contact

Further information and requests for resources should be directed to and will be fulfilled by the lead contact, Rosario Tomasello (tomasello.r@fu-berlin.de).

### Materials availability

This study did not generate new unique reagents.

### Data and code availability

• The data of the present study are available at: https://osf.io/quvtj/.
• The code used for analyzing the data is available at: https://osf.io/quvtj/.
• Additional information related to this study is available from the lead contact upon request.

## Acknowledgments

Research funding was provided by the 10.13039/100010663European Research Council, European Union, Advanced Grant “Material Constraints Enabling Human Cognition” (ERC-2019-ADG 883811), and the 10.13039/501100001659Deutsche Forschungsgemeinschaft, Germany Excellence Strategy cluster “Matters of Activity” (DFG EXC 2025/1).

## Author contributions

R.T. and F.P. conceived the study. R.T. and K.S. adapted the neural network model and ran the simulations. K.S. performed statistical analyses under R.T.’s supervision. F.D. carried out additional analyses, also supervised by R.T. R.T. and F.P. wrote the article, with contributions from K.S. and F.D.

## Declaration of interests

The authors declare no competing interests.

## Declaration of generative AI and AI-assisted technologies in the writing process

During the preparation of this work, the author(s) used ChatGPT5.0 for minor language suggestions and readability. After using this tool, the authors reviewed and edited the content as needed and take full responsibility for the content of the published article.

## STAR★Methods

### Key resources table

**Table:** 

REAGENT or RESOURCE	SOURCE	IDENTIFIER
**Software and algorithms**		

R (Version 2023.06.1 + 524)	R Core Team	R Core Team. (2020). R: A Language and Environment for Statistical Computing [Computer software]. R Foundation for Statistical Computing. https://www.R-project.org/.^110^
Brain-Constrained Neural Network	Felix Simulation Tool	Wennekers, T. (2009). Felix - A Simulation-Tool for Neural Networks (and Dynamical Systems). User Guide. https://pearl.plymouth.ac.uk/secam-research/1315.^111^ See also full model specification below and Tomasello, R., Garagnani, M., Wennekers, T., and Pulvermüller, F. (2018). A Neurobiologically Constrained Cortex Model of Semantic Grounding With Spiking Neurons and Brain-Like Connectivity. Frontiers in Computational Neuroscience *12*, 88. https://doi.org/10.3389/fncom.2018.00088.^51^ as well as, recently the model was implemented into the NEST simulator.^81^

**Deposited data**		

Project's repository	Open Science Framework	https://osf.io/quvtj/

### Method details

#### Full model specification

Each of the 12 simulated areas is implemented as two layers of artificial neuron-like elements (“cells”), 625 excitatory and 625 inhibitory, thus resulting in 15,000 cells in total. Each excitatory cell “*e*” consists of a leaky integrate-and-fire neuron with adaptation and simulates a single pyramidal cell representative of excitatory spiking activity in a cortical microcolumn, while its twin inhibitory cell “*i*” is a graded-response cell simulating the average inhibitory response of the cluster of interneurons situated in a local neighborhood.^112^^,^^113^ The state of each cell *x* is uniquely defined by its membrane potential *V*(*x,t*), specified by the following equation:

(Equation 1) $$\tau \cdot \frac{dV\left(x,t\right)}{dt}=-V\left(x,t\right)+k_{1}\left(V_{In}\left(x,t\right)+k_{2}\eta \left(x,t\right)\right)$$

where *V*_*In*_ (*x,t*) (defined by Equation 2) is the net input acting upon cell *x* at time *t* (sum of all inhibitory and excitatory postsynaptic potentials – I/EPSPs; inhibitory synapses are given a negative sign), *τ* is the membrane’s time constant, *k*_1_, *k*_2_ are scaling values (see Table below for the specific parameter values used in the simulations) and *η*(*e*,*t*) is a white noise process with uniform distribution over [-0.5,0.5]. Note that noise is an inherent property of each model cell, intended to mimic the spontaneous activity (baseline firing) of real neurons. Therefore, noise was constantly present in all areas, in equal amounts (inhibitory cells have *k*_2_ *=* 0, i.e., the noise is generated by the excitatory cells in the model for convenience).

(Equation 2) $$V_{In}\left(x,t\right)=-k_{G}\omega_{G}\left(A_{x},t\right)+\sum_{\forall y}w_{x,y}\cdot \phi \left(y,t\right)$$

In Equation 2, *y* is any other cell in the network, *w*_*x*,*y*_ is the weight of the link from *y* to *x*, and ϕ (*y*,*t*) is *y*’s current output (1 or 0), as defined below (3); ω_*G*_(*A*_*x*_,*t*) is the area-specific (or “global”) inhibition for area *A* where cell *x* is located (see explanation below and Equation 6): this term is identical for all excitatory cells *x* in *A* and absent for inhibitory cells (*k*_*G*_ is as scaling constant). The weights of inhibitory synapses are assigned a negative sign. The output (or transformation function) *ϕ* of an excitatory cell *e* is defined as follows:

(Equation 3) $$\phi \left(e,t\right)\left\{ \begin{array}{cc} 1 & \text{if}\;\left(V\left(e,t\right)-\alpha \omega \left(e,t\right)\right)>\;\text{thresh}\\ 0 & \text{otherwise}\; \end{array}\right.$$

**Table:** Model parameter values used in all networks during simulations

**Simulation Parameter Values**	

Time constant (excitatory cells)	τ = 2.5 (simulation time-steps)
Time constant (inhibitory cells)	τ = 5 (simulation time-steps)
Total input rescaling factor	*k*_1_ = 0.01
Noise amplitude	*k*_2_ = 3∗√(24/Δt)
Global inhibition strength	*k*_G_ = 0.73
Spiking threshold	*Thresh* = 0.18
Adaptation strength	ɑ = 7.0
Adaptation time constant	τ_*ADAPT*_ = 10 (time steps)
Rate-estimate time constant	τ_*Favg*_ = 30 (time steps)
Global inhibition time constant	τ_*GLOB*_ = 12 (time steps)
Sensorimotor input strength	*Inp* = 700

**Postsynaptic Membrane Potential Values**	

	*ϑ*_+_ = 0.15
	*ϑ*_--_ = 0.14

**Presynaptic Output Required for LTP**	

	*ϑ*_*pre*_ = 0.15
Learning Rate	Δ = 0.008

Thus, an excitatory cell *e* spikes (=1) whenever its membrane potential *V*(*e,t*) overcomes a fixed threshold *thresh* by the quantity *αω*.(*e,t*) (where *α* is a constant and *ω* is defined below). Inhibitory cells are graded response neurons as they intend to represent the average impact of a cluster of local interneurons; the output *ϕ*(*i,t*) of an inhibitory neuron *i* is 0 if *V*(*i,t*) < 0 and *V*(*i,t*) otherwise.

To simulate neuronal adaptation,^114^ function *ω*(*·,t*) is defined so as to track the cell’s most recent firing rate activity. More precisely, the amount of adaptation *ω*(*e,t*) of cell *e* at time *t* is defined by:

(Equation 4) $$\tau_{ADAPT}\cdot \frac{d\omega \left(e,t\right)}{dt}=-\omega \left(e,t\right)+\alpha_{adapt}\phi \left(e,t\right)$$

where *τ*_*ADAPT*_ is the “adaptation” time constant. The solution *ω*(*e,t*) of Equation 4 is the low-pass-filtered output *ϕ* of cell *e*, which provides an estimate of the cell’s most recent firing-rate history. A cell’s average firing activity is also used to specify the network’s Hebbian plasticity rule (see Equation 7, below); in this context, the (estimated) instantaneous mean firing rate *ω*_*E*_(*e*,*t*) of an excitatory neuron *e* is defined as:

(Equation 5) $$\tau_{Favg}\cdot \frac{d\omega_{E}\left(e,t\right)}{dt}=-\omega_{E}\left(e,t\right)+\phi \left(e,t\right)$$

Local (lateral) inhibitory connections and area-specific inhibition are also implemented, realizing, respectively, local and global competition mechanisms.^115^^,^^116^ More precisely, in Equation 2 the input *V*_*In*_(*x,t*) to each excitatory cell of the same area includes an area-specific (“global”) inhibition term *k*_*G*_.*ω*_*G*_(*e*,*t*) (with *k*_*G*_ a constant and *ω*_*G*_(*e*,*t*) defined below) subtracted from the total I/EPSPs postsynaptic potentials *V*_*In*_ in input to the cell; this regulatory mechanism ensures that area (and network) activity is maintained within physiological levels^74^:

(Equation 6) $$\tau_{GLOB}\cdot \frac{d\omega_{G}\left(e,t\right)}{dt}=-\omega_{G}\left(e,t\right)+\sum_{e\in \text{area}\;}\;\phi \left(e,t\right)$$

Excitatory links within and between (possibly non-adjacent) model areas are established at random and limited to a local (topographic) neighborhood; weights are initialized at random, in the range [0, 0.1]. The probability of a synapse to be created between any two cells falls off with their distance^74^ according to a Gaussian function clipped to 0 outside the chosen neighborhood (a square of size *n* = 19 for excitatory and *n* = 5 for inhibitory cell projections). This produces a sparse, patchy and topographic connectivity, as typically found in the mammalian cortex.^74^^,^^75^^,^^117^^,^^118^ The Gaussian function for within-area excitatory-excitatory connections has a center probability of 0.28, with σ_x_ = σ_y_ = 6.5, the Gaussian function for between-area excitatory-excitatory connections has a center probability of 0.15 with σ_x_ = σ_y_ = 4.5 and the one for inhibitory-excitatory connections has a center probability of 0.295, with σ_x_ = σ_y_ = 2.0.

The Hebbian learning mechanism implemented simulates well-documented synaptic plasticity phenomena of long-term potentiation (LTP) and depression (LTD), as implemented by Artola, Bröcher and Singer.^64^^,^^119^ In the model, we discretize the continuous range of possible synaptic efficacy changes into two possible levels, +Δ and −Δ (with Δ≪1 and fixed). Following Artola et al., we defined as “active” any (axonal) projection of excitatory cell *e* such that the estimated firing rate *ω*_*E*_(*e*,*t*) of cell *e* at time *t* (see Equation 5) is above Θ_pre_, where Θ_pre_∈[0,1] is an arbitrary threshold representing the minimum level of presynaptic activity required for LTP to occur. Thus, given a pre-synaptic cell *i* making contact onto a post-synaptic cell *j*, the change Δ*w*(*i,j*) efficacy of the (excitatory-to-excitatory) link from *i* to *j* is defined as follows:

(Equation 7) $$\Delta w\left(i,j\right)=\left\{ \begin{array}{ccc} +\Delta & {if\;\omega_{E}\left(i,t\right)\geq \theta_{pre}\;and\;V\left(j,t\right)\geq \theta }_{+} & \left(LTP\right)\\ -\Delta & {if\;\omega_{E}\left(i,t\right)\geq \theta_{pre}\;and\;\theta_{-}\leq V\left(j,t\right)\;<\;\theta }_{+} & \left(homosynaptic\;LTD\right)\\ -\Delta & {if\;\omega_{E}\left(i,t\right)<\;\theta_{pre}\;and\;V\left(j,t\right)\geq \theta }_{+} & \left(heterosynaptic\;LTD\right)\\ 0 & otherwise & \end{array}\right.$$

#### Neural network initialisation and input patterns

To simulate initial perceptual learning of fine-grained color categories and spoken word forms, 16 networks were initially created. Each network was initialised by determining the presence and weight of between-neuron connections using an algorithm that realises sparse and initially weak random links with a neighborhood bias (for a detailed explanation, see^76^). Connections were created within each area (among e-cells, among i-cells, and between these) and between the e-cells of next-neighbour areas and some areas whose biological correlates are interlinked in the human brain. In order to model the emergence of color category learning, we created sets of sensorimotor stimulation patterns that included 22 active neurons, each thought to represent key perceptual information about shades of the same color (two shades of blues and greens) and phonological word forms. These active neurons per pattern were selected from a pool of 625 e-cells in a primary area (constituting 3.5% of the total cells in an area).

The visual color patterns were constructed with an inherent similarity structure in their neural representations. Specifically, between the input pattern of the two shades of blue and the two shades of green, 22,8% of the neurons were shared, while a smaller proportion (5%) of the neurons were shared between the blue and green sensorimotor pattern (Figure 2B), to capture the general features they may have in common (i.e., belonging to the same overarching color domain; see introduction section). The three-word forms or labels were also encoded through sensorimotor patterns that included an auditory and an articulatory activation pattern. However, each input was composed of 22 cells randomly selected from the 625 e-cells and did not exhibit any structural similarities between them (Figure 2B). These auditory and articulatory patterns are thought to represent different acoustic and articulatory word forms. Entirely different word form patterns were chosen to ensure that any observed differences would not be due to any similarities between the label patterns. It is important to note that inputs to the human brain, especially in the realm of color perception, are inherently complex. In the visual cortex, specialised neurons are selectively tuned to specific wavelengths, contrasts, and hues, allowing for fine-grained color discrimination through distributed population coding.^103^^,^^104^^,^^120^^,^^121^ Our model did not aim to replicate these intricate encoding mechanisms or to generate input patterns based directly on colourimetry parameters such as wavelength or luminance. Instead, the neural network was designed to capture an abstract but biologically grounded representation of perceptual similarity, modeling how similar colors activate overlapping populations of feature-selective neurons. Each color input was encoded as a distributed activation pattern across visual units, with neighboring colors (e.g., light and dark blue) sharing a subset of activated neurons, while distinct colors (e.g., blue vs. green) showed very low overlap. This approach allowed us to investigate how linguistic labeling modulates the internal neural representations of perceptually similar stimuli.

While each of the 16 networks had its own initialisation of synaptic links that were sparse and random, the to-be-learned input patterns representing colors and word forms described above were the same for all the networks. This was meant to imitate the fact that different language users share the same word forms and concepts characterised by the same phonological, perceptual and semantic features. The 16 networks were considered to simulate the brains of different language learners, which are all similar structurally and functionally, as they occur in similarly structured brains and are governed by the same neurobiological principles but still differ across individuals.

#### Simulated learning procedures

##### Initial perceptual color learning and phonological word forms phase

In this learning phase, the model was used to simulate the perceptual learning of two different shades of blue and green, respectively. Perception of light and dark colors was simulated with color input patterns to the primary visual (V1) region, while all three non-relevant primary areas (M1i, A1, and M1L) received variable noise inputs that changed at each learning event. The variable noise reflects uncorrelated inputs to these areas, which are typically present during learning and appear to be critical for preventing excessive cell assembly growth into adjacent regions.^49^^,^^80^^,^^122^ To simulate babbling and label acquisition, pairs of word-form sensorimotor inputs were presented simultaneously in the auditory (A1) and articulatory (M1i) regions, mimicking the neural articulatory and acoustic activity typically activated in these regions during word production.^68^^,^^69^^,^^70^ Also, when simulating word form production, the other primary areas (V1 and M1L) received the variable noise input. Overall, this perceptual simulation phase simulated a child that, in certain learning instances, encounters the blue and green colors (like light blue skies or darker blue seas, and different greens found in nature), and in other instances the production of meaningless phonological word forms (label) prior to a combination thereof.

For each training trial, the color and word form inputs were presented to the model's primary area(s) for 16 simulation time steps with an interstimulus interval of 30 simulation time steps or until the global inhibition in area V1, TO, AT, and PFL was below a threshold of 0.55. The color and word form patterns were presented separately, each in 1000 trials whose sequence was randomised. Prior to the presentation of each pattern, a global network reset was carried out in which the membrane potential of all e-and i-cells was set to 0. This reset ensured that the neural activity from the previously presented pattern would not influence the subsequent one during the learning process. In addition, a reset was implemented to prevent the formation of distinct neural circuits from merging (see Sec. below on how cell assemblies were identified), which is common if only a very small number of representations is learned in this kind of model. However, importantly, the procedure adopted did not interfere with learning or the formation of neural circuits across the modeled regions.

##### Semantic learning phase of color terms in Russian and English speakers

After the networks were exposed to perceiving colors and producing word forms separately, the same networks entered a second learning phase. Specifically, the same 16 networks, which had previously been trained on colors and word forms (1000 learning trials each) before semantic learning, were duplicated to create 16 English and 16 Russian networks. This ensured that the learned colors and word form patterns, as well as the synaptic weights, were the same in both language-specific models. The only between-model difference was the way in which color and word forms were co-presented in semantic learning. To this end, the association between learned color patterns and word forms varied based on whether the simulation mimicked English or Russian color naming systems (i.e., blue and green colors). Specifically, in the English model, both blue and green shades shared an identical label. Conversely, in the Russian model, each blue shade was paired with a distinct label, while both green shades shared a single label. Specifically, the model was stimulated by activating neurons corresponding to color and word form patterns previously learned in the primary visual cortex (V1) for color perception, and in both perisylvian primary regions (A1 and M1i) for word form production. Additionally, variable uncorrelated inputs were presented to the non-relevant modality-preferential area (M1L, Figure 2C). This approach aimed to simulate the color naming system for blues and greens of English and Russian speakers, by mimicking learning scenarios where a person is exposed to colors while at the same time uttering the labels that refer to it. This process mimics direct associative learning, as documented for object- and action-related words.^123^^,^^124^

The learning procedure for each trial mirrored that of the first learning phase: Inputs were provided for 16 simulation time steps, and the network was reset before each pattern presentation (for further details, refer to the section above). Overall, each pair of color and label was presented in 1000 randomised learning trials. Therefore, there were 4,000 learning trials for each model, with 1,000 presentations of each color shade, but 2,000 presentations of most verbal labels (except for the designations of Russian blues, which had 1,000). The number of learning trials was selected based on prior simulation studies, demonstrating that prolonged training does not significantly alter the size or dynamics of neural circuits.^44^^,^^48^^,^^51^^,^^80^^,^^93^^,^^125^

#### Simulation of neurophysiological indices of color perception

After completing the training phase, the neural network was used to simulate color perception and recognition in both Russian and English neural models, while recording neurophysiological brain responses (spike activity) over time. The objective was to replicate the experimental procedure in color perception from a previous study.^19^ To achieve this, each learned pattern for the color shades (blue and green) was presented as input to the primary visual area (V1), simulating color perception in different speakers. The stimulation lasted for 2 time steps, followed by a 28 time steps period without additional input. A baseline value was recorded during the 10 time steps preceding stimulation. After each trial, the network was reset by setting the membrane potential of all e− and i-cells to 0, consistent with the procedure used in the learning phases.

### Quantification and statistical analysis

For each model type (Initial exposure phase, Russian and English models), an initial set of 16 networks underwent learning (in total, 48 networks). However, only 13 networks per model type entered the statistical analysis (in total, 39 networks). The exclusion of three networks per model type was due to some merging of cell assemblies (CAs) within these networks, leading to the co-activation of multiple assemblies and excessive activation spread during either the English or Russian simulations. This can be considered to reflect three subjects that failed to learn novel word-referent mapping in a learning experiment due to not paying attention or not following the task. Also, by excluding these networks, the analysis focused on models that successfully learned distinct mappings, ensuring the robustness and validity of the findings.

#### Cell assembly definition and extraction

To identify the cell assemblies (CAs) that emerged across the modeled areas during both learning phases, we reactivated CA circuits by simulating color perception by stimulating the V1 area with a learned color pattern for 16 simulation steps. During the extraction, no learning occurred during stimulation. No uncorrelated patterns were given to the other primary regions (M1L, A1, and M1i), to capture the cells formally belonging to a CA, following the same procedure as in previous simulation work.^47^^,^^51^^,^^76^^,^^80^

For each modeled region, we determined the peak firing rate observed across all 625 excitatory cells within that region (in total 7,500 e-cells) during the 30 time steps post-stimulation. A cell was designated as part of a cell assembly if, at any given time step, its firing rate achieved a minimum of 20% of the rate of the most responsive cell in that region (assuming the peak firing rate was at least 0.2). In estimating a cell’s average firing rate, we utilised the value *w*_*E*_(*e*,*t*) derived from Equation 4, incorporating a time-constant *τ*_*Favg*_ = 5. A specific excitatory cell (e-cell) was considered part of a given cell assembly (CA) circuit only if its time-averaged rate (referred to as ”firing rate“) surpassed a threshold *θ*, which was both area- and input-pattern dependent. This threshold was defined as *γ* times the maximal time-averaged response of a single cell within that area to pattern *w*. More formally,

$$\theta =\theta A\left(w\right)=\gamma \;\max_{x\in A}\bar{O{\left(x,t\right)}_{w}}$$

where $\bar{O{\left(x,t\right)}_{w}}$ is the estimated time-averaged response of cell *x* to word pattern *w* (see Equation 6 in STAR Methods) and *γ*∈[0,1] is a constant (we used *γ* = 0.5 on the basis of previous simulation results^53^^,^^76^^,^^93^). This was computed for each of the 16 trained network instances and the estimated mean firing rate for each excitatory neuron in the model in response to each stimulation pattern. Mean firing rate in the 30-time steps past stimulation were also converted into activation vectors submitted to Representational Similarity Analysis (RSA^82^), for calculating dissimilarity matrices. The extracted CAs were further subdivided into ‘unique’ and ‘shared’ neurons (see Section below).

#### Representational similarity analysis (RSA)

After cell assembly extraction, during which the average firing rate of all neurons in each region across various models was determined in response to specific input patterns, we generated representational activation vectors for each stimulation and region. These vectors were constructed using the unthresholded activation values of all 625 neurons. Activation vectors following visual stimulations (by light/dark blues/green) were calculated for both the Russian and the English models. Euclidean distances between these vectors were calculated and plotted into 4 x 4 “representational dissimilarity matrices (RDMs)” illustrating the degree of difference between the corresponding neural representations^82^ (see also for previous simulation procedure^48^^,^^126^). This analysis was specifically performed to assess the level of (dis)similarity in the network’s responses to the four color patterns (two shades of blue and two shades of green) following perceptual learning and semantic learning. The dissimilarity values were initially computed for each network and region separately and then averaged across the 13 networks for each model type. The matrices were generated for the visual, auditory, and articulatory systems and the central hub regions, as well as for all relevant network regions collapsed (see Figure 3A), excluding the two lateral motor regions (PM and M1L). These regions were excluded due to their minimal neural activations, attributed to the absence of direct input. The data visualisation was conducted in RStudio using the “lattice” package.^127^

To assess any differences in dissimilarity (Euclidean distance) between the neural representations of blue and green color shades in the English and Russian models, we conducted a first 2-way Statistical Analysis of Variance (ANOVA) on all relevant regions collapsed. The ANOVA included the factors Model Type (2 levels: English and Russian) and Color (2 levels: Blue and Green). The same two-way ANOVA was run for the four central hub regions (AB, AT, PFL, PFI) collapsed together, as well as for the visual AT hub alone. The latter analysis on the hub regions and AT alone was to ensure that the findings were not influenced by the direct input structure of the primary regions of the neural network.

Subsequently, to explore any changes in color representation within the visual system (V1, TO, and AT combined) before and after symbolic learning, two separate 2-way ANOVAs were conducted by contrasting English and Russian model types with the initial exposure model. The ANOVA included factors Learning Phase (2 levels: perceptual and either English or Russian color term learning) and Color (2 levels: Blue and Green). A similar analysis was carried out specifically for the central visual (AT) regions for a more detailed examination. Note that for the no-label vs. Russian and English label conditions, we did not perform an analysis collapsing all relevant regions together as above, as the neural color representations for the no-label conditions were restricted within the visual system. Greenhouse–Geisser correction^128^ was applied when sphericity violations were found. Corrected *p*-values are reported throughout. We conducted these analyses in RStudio with the assistance of the ”rstatix“ package,^129^ and for visual presentations, we used the “ggplot2” package.^130^

#### Neuron type (shared vs. unique) calculation & analysis

To gain a deeper understanding of any differences between the neural representations activated by different colors in the different network types, we followed approaches by.^47^^,^^48^ This involved analysing whether the neurons within the cell assemblies activated by a specific color pattern were unique or instance-specific to that pattern or shared with cell assemblies activated by other patterns. To this end, for each color stimulus, we counted the number of cell assembly neurons in each model area that were activated by both the dark and light version of a given color ('shared neurons') or just by one shade of that color exclusively ('unique neurons'). The calculations were initially performed for each of the 13 neural networks’ regions per model type (following perceptual learning and for both the English and Russian models). The results from these individual network analyses were then aggregated. In the final results, we present both the absolute counts of shared and unique neurons and the percentage of shared neurons, calculated relative to the total number of cells comprising a cell assembly within a given region.

To examine the neural representations after perceptual learning, the neural distributions and density of neuron types (subdivided into either shared or unique neurons) across areas were plotted. The same statistical test was once again performed after semantic learning and compared between English and Russian models. To this end, we conducted 3-way ANOVAs with the factors Model Type (English and Russian), Color (2 levels: Blue and Green), and Neuron Type (2 levels: Shared and Unique). Subsequently, to examine the representational difference within each model type, Russian and English, separately, a further two-way ANOVA was run with the factors Color (2 levels: Blue and Green) and Neuron Type (2 levels: Shared and Unique). These ANOVAs were run on all relevant model regions combined (i.e., 10 regions, see above), the four hub regions (AT, PFL, PFI, PB) aggregated together, and on visual hub AT separately, similarly to the analysis run for the RDMs above.

In an additional analysis to examine neural changes induced by distinct labeling within the visual system, we compared the neural representations related to color words in the English and Russian models with the same model that had undergone only the color perceptual simulation prior to labeling association (see above for more details). To achieve this, we conducted separate 3-way ANOVAs for the Russian and English models compared to the perceptual color phase, with factors including Label (2 levels: post-perceptual learning vs. post-semantic learning), Color (2 levels: Blue vs. Green), and Neuron Type (2 levels: Shared vs. Unique). Initially, we analyzed data collapsed across the three visual regions (V1, TO, and AT), followed by more focused analyses on the AT region specifically. These ANOVAs were performed on the absolute number of shared and unique neurons within the visual system that were activated by color input patterns, as color neural representations emerged primarily within the visual system during the color perceptual phase.

#### Neurophysiological indices of color perception - Calculation and analysis

During the color recognition process, we meticulously recorded the area-specific ‘within-cell assembly (CA) activity’ resulting from color input patterns for every simulation time step, encompassing the 10 time steps preceding stimulus onset and the 28 time steps following stimulus offset. Specifically, we classified responsive neurons as belonging to the cell assemblies if their estimated firing rate reached at least 20% of the maximal firing rate in their area, aligning with the CA extraction procedure described above. Responsive neurons were identified and classified as exhibiting either ‘unique’ or ‘shared’ activation by comparing spiking patterns for shades of the same color (blue or green) over time (see supplemental information). To isolate the unique neural response to either the dark or light shades, we subtracted the shared neural response from the total neural responses specific to each shade of the same color. This calculation can be expressed as:

$${}_{\text{Unique}}R_{\text{dark}}=\left({\text{Rtot}}_{\text{dark}}-\text{R}\mathrm{shared}\right)$$

Where _*Unique*_*R*_*dark*_ represents the network neural response of unique neurons specific to the dark shade over the light one, *Rtot*_*dark*_ is the total number of spikes in neurons responsive to the dark shade, and R*shared* represents the number of spikes in neurons that are part of the CAs of both dark and light shades. Calculating the subtraction allows us to isolate the responses of unique neurons specifically activated by dark shades.

To evaluate the network’s ability to distinguish between different color shades, we analyzed the activation of unique neurons specific to each shade. This approach serves as a computational analogue to the neural responses observed in the color mismatch negativity (MMN),^19^ which reflects the brain’s sensitivity to unexpected changes in color shades. Stronger MMN responses were observed when different color shades were associated with distinct labels, reflecting the lexical distinction at the neural level. This effect might be driven by instance-specific (unique) neural representations tied to each distinct color term during color perception. To this end, to assess significant differences in _*unique*_*R* neural responses over time between the Russian and English models for the two color types, we conducted a two-way ANOVA with factors Model Type (Russian and English) and Color (Blue and Green) averaged across 10 simulation timesteps from stimulation offset (2–12 simulation steps). We report *p*-values that were adjusted for multiple comparisons using the Greenhouse-Geisser correction.^128^
