# Supplementary material for: How language modulates color perception in a brain-constrained deep neural network
Source: iScience. 2026 Jan 29;29(3):114832. doi: 10.1016/j.isci.2026.114832 (PMC12927276; doi:10.1016/j.isci.2026.114832)
Supplement: Document S1. Figure S1 and Tables S1–S3 [file mmc1.pdf]

iScience, Volume *29*

## Supplemental information

# How language modulates color perception

# in a brain-constrained deep neural network

**Rosario Tomasello, Kai Shaman, Fynn R. Dobler, and Friedemann Pulvermüller**

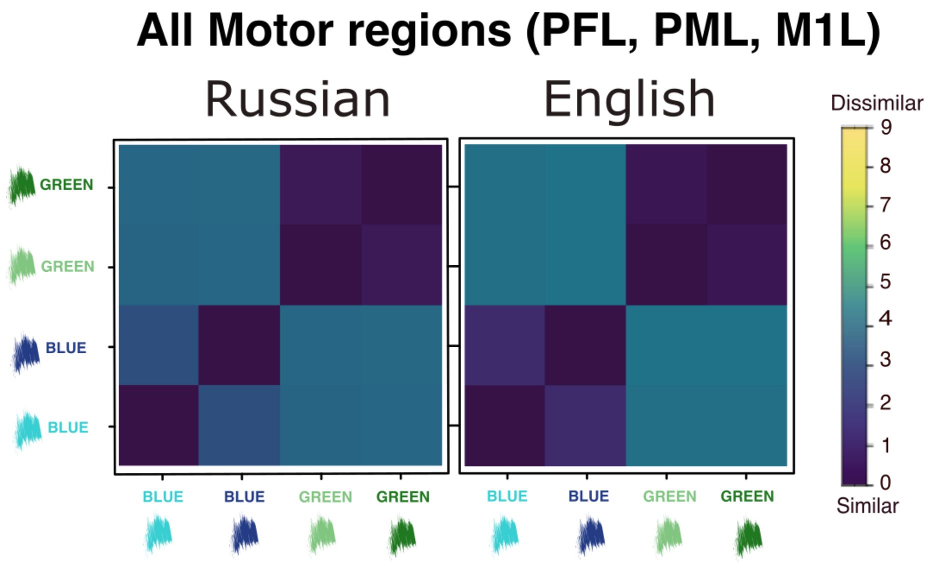


**Figure S1: Results of Representational Dissimilarity Matrix (RDMs) in the modelled motor regions.**

The panel shows the RDMs matrices for the Russian model and the English model for the motor regions collapsed together. Each RDM is a 4x4 matrix, where each square represents, through color coding, the dissimilarity between the respective pairs of the four color patterns. The dissimilarity measure used was the Euclidean distance, with 0 indicating similar neural representations. Note, however, that neural representations, particularly in secondary (PML) and primary (M1L) motor regions, were very low, with only a small number of cells contributing to the resulting neural circuits.

**Table S1: Connectivity Structure of the brain-constrained neural network.**

This table presents the references used to establish the connectivity structure of the network model, categorized by connection type and the regions involved. Taken from Tomasello et al., 2018.

| **Between-area connectivity** (black arrows) | |
|---|---|
| **Modelled Areas** | **References** |
| *Perisylvian system* | |
| A1, AB, PB | (Kaas and Hackett, 2000; Pandya, 1995; Rauschecker and Tian, 2000) |
| $PF_i$, $PM_i$, $M1_i$ | (Pandya and Yeterian, 1985; Young et al., 1994) |
| | |
| *Extrasylvian system* | |
| V1, TO, AT | (Bressler et al., 1993; Distler et al., 1993) |
| $PF_L$, $PM_L$, $M1_L$ | (Arikuni et al., 1988; Dum and Strick, 2005, 2002; Lu et al., 1994; Pandya and Yeterian, 1985; Rizzolatti and Luppino, 2001) |
| *Between system* | |
| AT, PB | (Gierhan, 2013) |
| $PF_i$, $PF_L$ | (Yeterian et al., 2012) |
| **Long distance cortico-cortical connections** (purple arrows) | |
| *Perisylvian system* | |
| $PF_i$ , PB | (Catani et al., 2005; Makris and Pandya, 2009; Meyer et al., 1999; Parker et al., 2005; Paus et al., 2001; Rilling et al., 2008; L. M. Romanski et al., 1999) |
| *Extrasylvian system* | |

| | |
|---|---|
| AT, $PF_L$ | (Bauer and Jones, 1976; Chafee and Goldman-Rakic, 2000; Eacott and Gaffan, 1992; Fuster, 1995; Parker and Gaffan, 1998; Ungerleider et al., 1989; Webster et al., 1994) |
| *Between system* | |
| PB, $PF_L$ | (Pandya and Barnes, 1987; L.M. Romanski et al., 1999; L. M. Romanski et al., 1999) |
| AT, $PF_i$ | (Pandya and Barnes, 1987; Petrides and Pandya, 2009; Rilling, 2014; Romanski, 2007; Ungerleider et al., 1989; Webster et al., 1994) |
| **Second-next-neighbor "jumping" links** (blue arrows) | |
| *Perisylvian system* (Rilling et al., 2012, 2008; Thiebaut de Schotten et al., 2012) | |
| A1, PB | (Pandya and Yeterian, 1985; Young et al., 1994) |
| PB, PMi | (Rilling et al., 2008; Saur et al., 2008) |
| AB, PFi | (Kaas and Hackett, 2000; Petrides and Pandya, 2009; Rauschecker and Scott, 2009; L.M. Romanski et al., 1999) |
| PFi, M1i | (Deacon, 1992; Guye et al., 2003; Young et al., 1994) |
| *Extrasylvian system* (Thiebaut de Schotten et al., 2012) | |
| V1, AT | (Catani et al., 2003; Wakana et al., 2004) |
| AT, $PM_L$ | (Bauer and Fuster, 1978; Chafee and Goldman-Rakic, 2000; Fuster et al., 1985; Pandya and Barnes, 1987; Seltzer and Pandya, 1989) |
| TO, $PF_L$ | (Bauer and Jones, 1976; Fuster et al., 1985; Fuster and Jervey, 1981; Makris and Pandya, 2009; Seltzer and Pandya, 1989) |
| $PF_L$, $M1_L$ | (Deacon, 1992; Guye et al., 2003; Young et al., 1994) |

**Table S2: Additional statistical results of the Representational dissimilarity analysis (RDMs)**

We report below the results of additional analyses on the dissimilarity values between neural representations of blue and green color shades in the English and Russian models within the anterior temporal region (AT). First, a two-way ANOVA was conducted with the factors Model Type (English vs. Russian) and Color (Blue vs. Green) after symbolic learning. Second, to assess changes across learning phases, two separate 2×2 ANOVAs were performed for the English and Russian models, respectively. Each included the factors Learning Phase (perceptual learning vs. symbolic learning in either English or Russian model) and Color (Blue vs. Green).

**Visual Hub (AT) Region - 2x2x2 ANOVA (Model Types X Color)**

| Factor | F-Statistic | p-value |
|---|---|---|
| Model Type | 46.4 | < .001 |
| Color | 33.4 | < .001 |
| Model Type:Color | 56.9 | < .001 |

**Visual Hub (AT) Region - 2x2x2 ANOVA (Learning phase X Color)**

*Russian vs initial perceptual model*

| Factor | F-Statistic | p-value |
|---|---|---|
| Learning phase | 0.95 | = .347 |
| Color | 39.4 | < .0001 |
| Learning phase:Color | 155.3 | < .0001 |

*English vs initial perceptual model*

| Factor | F-Statistic | p-value |
|---|---|---|
| Learning phase | 40.5 | < .001 |
| Color | 5.0 | = .044 |
| Learning phase:Color | 3.6 | = .079 |

**Table S3: Additional statistical results on the Neuron Types (Shared vs. Unique) results**

Here, we report the results of analyses on microstructural changes in shared and unique neuron types across the two model types, as well as in comparison to the initial perceptual model, within the anterior temporal visual hub (AT). A 2×2×2 ANOVA was conducted with the factors Model Type (English, Russian), Color (Blue, Green), and Neuron Type (Shared, Unique). To further investigate representational differences within each model type separately, additional 2×2 ANOVAs were run with the factors Color (Blue, Green) and Neuron type (Shared, Unique). Moreover, we conducted separate 2×2×2 ANOVAs for each model type (English and Russian), comparing them to the perceptual learning phase. These included the factors Learning phase (Post-Perceptual vs. Post-Semantic), Color (Blue, Green), and Neuron type (Shared, Unique).

**Visual Hub (AT) Region - 2x2x2 ANOVA (Model Type X Color X Neuron Type)**

| **Factor** | **F-Statistic** | **p-value** |
|---|---|---|
| Model type | 11.7 | = .005 |
| Color | 0.50 | = .489 |
| Neuron type | 21.4 | < .001 |
| Model type:Color:Neuron type | 75.9 | < .001 |

**Visual Hub (AT) Region - 2x2 ANOVA (Color X Neuron type)**

*Russian model*

| **Factor** | **F-Statistic** | **p-value** |
|---|---|---|
| Color | 6.20 | = .028 |
| Neuron type | 0.57 | = .465 |
| Color:Neuron type | 175.4 | < .0001 |

*English model*

| **Factor** | **F-Statistic** | **p-value** |
|---|---|---|
| Color | 0.79 | = .391 |
| Neuron type | 60.7 | < .001 |
| Color:Neuron type | 1.1 | = .311 |

**Visual Hub (AT) Region - 2x2 ANOVA (Model type X Color)**

*Unique Neurons*

| **Factor** | **F-Statistic** | **p-value** |
|---|---|---|
| Model Type | 38.99 | < .001 |
| Color | 42.85 | < .001 |
| Model Type:Color | 50.87 | < .001 |

*Shared Neurons*

| **Factor** | **F-Statistic** | **p-value** |
|---|---|---|
| Model type | 53.34 | < .001 |
| Color | 15.87 | = .002 |
| Model type:Color | 80.12 | < .0001 |

**Visual Hub (AT) region - 2x2x2 ANOVA (Learning phase X Color X Neuron type)**

*Russian vs initial perceptual model*

| **Factor** | **F-Statistic** | **p-value** |
|---|---|---|
| Learning phase | 157.78 | < .0001 |
| Color | 2.12 | =.170 |
| Neuron type | 36.6 | < .0001 |
| Learning phase:Neuron type:Color | 152.27 | < .0001 |

*English vs initial perceptual model*

| **Factor** | **F-Statistic** | **p-value** |
|---|---|---|
| Learning phase | 164.2 | < .0001 |
| Color | 0.56 | = .469 |
| Neuron type | 0.054 | = .821 |
| Learning phase:Neuron type | 161.2 | < .0001 |
